# Cardiac involvement of the systemic disorder *myotonic dystrophy type II *- detection by CMR

**DOI:** 10.1186/1532-429X-16-S1-P390

**Published:** 2014-01-16

**Authors:** Luisa Grosse, Julius Traber, Ulrike I Grieben, Wolfgang Utz, Matthias A Dieringer, Peter Kellman, Simone Spuler, Jeanette Schulz-Menger

**Affiliations:** 1 Working Group on Cardiovascular Magnetic Resonance, Experimental and Clinical Research Center a joint cooperation between the Charité Medical Faculty and the Max-Delbrueck Center for Molecular Medicine HELIOS Klinikum Berlin Buch, Department of Cardiology and Nephrology, Berlin, Germany; 2 Muscle Research Unit, Experimental and Clinical Research Center a joint cooperation between the Charité Medical Faculty and the Max-Delbrueck Center for Molecular Medicine, Berlin, Germany; 3 Laboratory of Cardiac Energetics, National Institutes of Health/NHLBI, Bethesda, Maryland, USA

## Background

Myotonic dystrophy type II (formerly denoted as proximal myotonic myopathy (PROMM)) is an autosomal dominantly inherited disease [1]. Sufferers are afflicted with skeletal muscle (SM) symptoms. Histopathologic changes of the SM include mild fibrosis and fatty degeneration[2]. The multisystemic disorder is also characterized by endocrine and metabolism disorder such as hypercholesterolemia and diabetes mellitus type II as well as cardiac arrhythmias [1]. The aim of this study is to evaluate myocardial structural abnormalities in preserved ejection fraction (EF).

## Methods

We prospectively enrolled 28 subjects (age 53.1 ± 10.7 y, 20 women) with a genetically confirmed diagnosis of PROMM. The criteria for exclusion were known cardiac diseases and contraindication for CMR. We assessed biplanar left-ventricular (LV) volumes, mass and function applying cine imaging (TR 247.42 ms, TE 1.14 ms, FOV 340 ms, matrix 192×192, slice thickness 6 mm) using a 1.5 T Scanner (Avanto, Siemens Healthcare, Germany, 12 channel surface coil). Late enhancement imaging (LGE) (GRE, TR 800 ms, TE 5.02 ms, FOV 350 ms, matrix 256×256, slice thickness 7 mm) was performed to detect myocardial fibrosis about 10 minutes after injection of gadoteridol (0.2 mmol/kgbw). Furthermore, we used a previously described fat/water sequence[3] (multi-echo GRE, 4-echos, TE 1.53-8.42 ms, FOV 360 mm, matrix 256×256, slice thickness 6 mm) to identify myocardial fat deposits. Data were analyzed using cvi^42 ^(circle cardiovascular imaging Inc., Canada).

## Results

**Figure 1:**
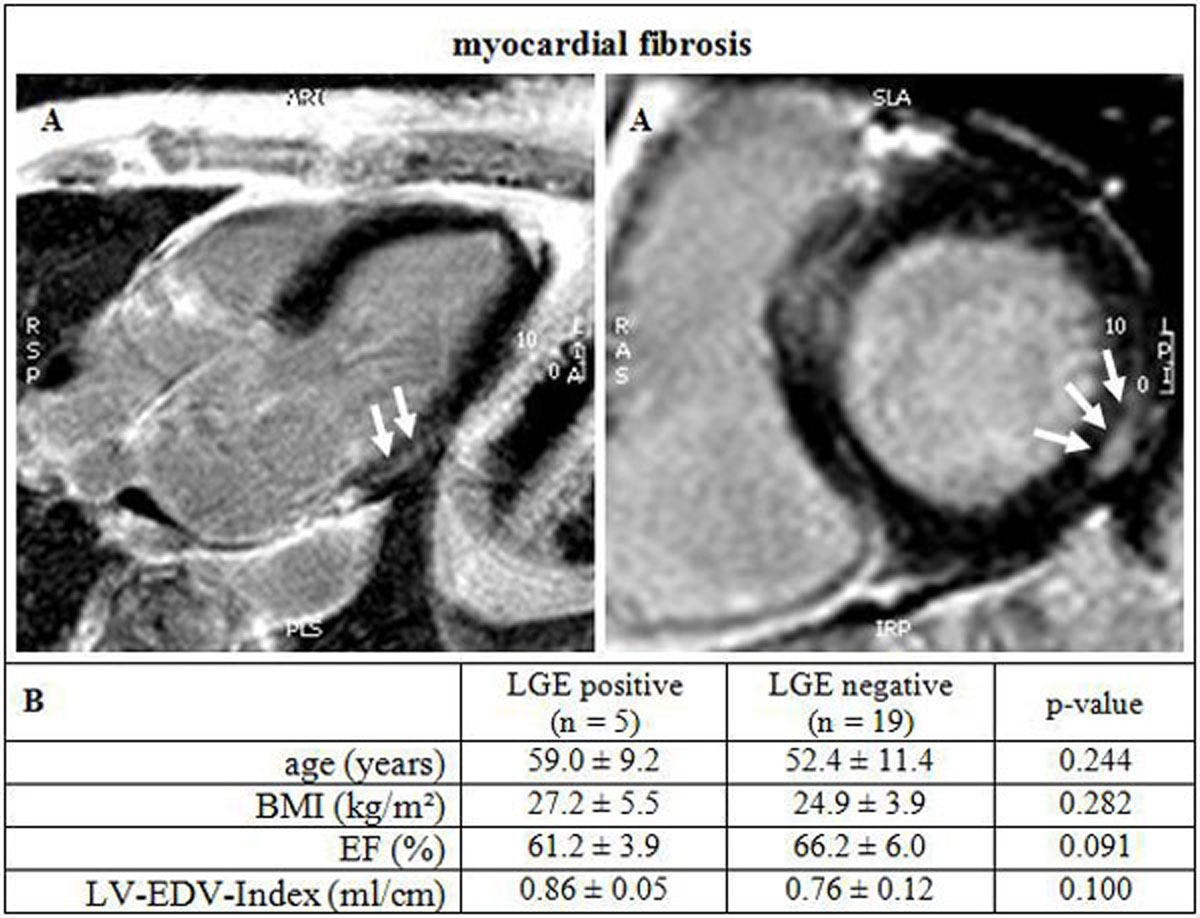
**(A) Detected subepicardial LGE (arrows) in a patient with PROMM**. (B) Comparison of LGE positive to negative subjects. BMI: Body mass index, EF: ejection fraction, LV-EDV-Index: left-ventricular-enddiastolic volume-index.

**Figure 2:**
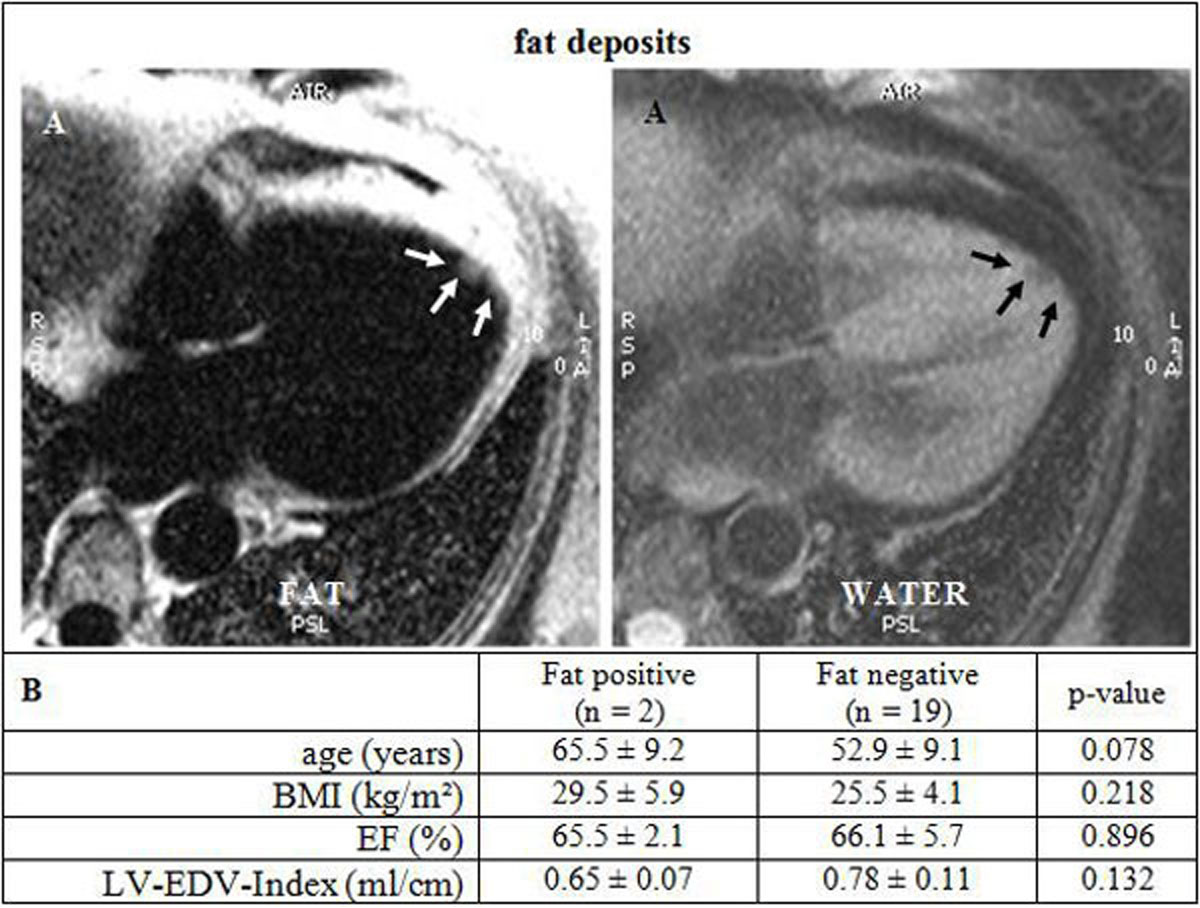
**(A) Indicated fat deposit (arrows) in a patient with PROMM**. The view shows both the water and the fat-seperated image. (B) Comparison of the subjects who have fat deposits with those who have no detected myocardial fat. BMI: Body mass index, EF: ejection fraction, LV-EDV-Index: left-ventricular-enddiastolic volume-index.

24 data sets were completed (age 53.8 ± 10.9 y, EF 65.2 ± 5.8%, 17 women). All applied sequences were evaluable besides 3 fat/water images due to artifacts. None of the patients had wall motion abnormalities. Myocardial fibrosis were noticeable in 5 of the 24 subjects (2 women). No significant differences of age (p = 0.24) and LVEF (p = 0.09) were found between positive and negative LGE (Figure 1). These fibrous changes were mostly localized subepicardial inferolateral basal. No fat deposit was found in this region (Figure 1). Interestingly, small myocardial fat deposits were identified in the apical portion of the interventricular septum in 2 patients (Figure 2).

## Conclusions

To the best of our knowledge this is the first systematic CMR study in patients with PROMM. Although all patients had preserved LVEF we could detect myocardial fibrosis in one fifth of all patients, suggesting CMR is feasible to detect such subclinical myocardial manifestations. Interestingly, we could also detect fat deposits. Further studies to look for diffuse alterations and correlations to clinical events should come next.

## Funding

University funding hold by Schulz-Menger, J.
